# Association of occlusal wear facets in patients with temporomandibular disorders

**DOI:** 10.6026/973206300161060

**Published:** 2020-12-31

**Authors:** Faazila Fathima, Sreedevi Dharman, P Senthil Murugan

**Affiliations:** 1 Saveetha Dental College, Saveetha Institute of Medical and Technical Sciences, Saveetha University, Chennai 77, India

**Keywords:** Bruxism, clenching, occlusal wear, temporomandibular disorder

## Abstract

Occlusal changes were important factors in temporomandibular disorder (TMD). It is of interest to evaluate the association of occlusal wear facets in TMD patients. We used a dataset of 49 patients with and without TMD for this study. Occlusal wear facets were evaluated using Smith and Knight tooth wear index. Data shows that teeth wear was present more in patients with TMD (55%). The age group 26-40 years showed high prevalence of teeth wear (grade1) in TMD patients (P value = 0.034). TMD was present more in females than males. Female (54%) patients with TMD showed more teeth wear compared to males. Most patients with TMD showed posterior teeth wear (61%) than generalized teeth wear. Thus, association was present between occlusal teeth wear and TMD patients especially in the age group of 26-40 years. Hence, proper evaluation of occlusal factors will aid in early diagnosis of TMDs.

## Background:

TMD is a functional and pathological condition affecting temporomandibular joint, the masticatory musculature, or both in the maxillofacial region [1]. Approximately 33% of the population has at least one TMD symptom and 3.6% to 7% of the population has TMD with more severity to cause them to seek treatment [2]. Etiology of TMD is multifactorial, and is related to a number of dental and medical conditions, such as occlusion, posture, parafunctional habits, restorative procedures, orthodontic treatment, emotional stress, trauma, anatomy of the disc, pathophysiology of the muscles, genetic and psychosocial factors such as age, and gender [3]. Typical signs and symptoms of TMD are pain, limited mouth opening, joint sounds, mandibular deviation, and chewing disability. These symptoms may exist alone or in combination. However, it cannot be stated that these factors are predisposing TMD or are only coincidental [4]. Due to the etiological complexity and variety of signs and symptoms that may represent other pathologies, recognition and differentiation of TMDs is not very clear to the professional. Therefore, routine screening should be combined with the anamnesis and selective clinical examination, for the professional to perform an accurate diagnosis and therefore develop the proper treatment plan [5]. Teeth wear is considered as a visible sign of physiological functional wear and a rather rapid onset in bruxism. It is probably seen more as a result of functional disturbance in the masticatory system [6]. Mechanical tooth surface wear is caused by harmful or parafunctional mandibular movements. Parafunctional habits such as bruxism, tooth clenching, gum chewing, biting foreign objects, and prolonged nail biting might increase the risk of developing TMD [7]. Parafunctional activities are usually harmless, until the forces exerted exceed the structural tolerance [8]. Bruxism and clenching reportedly lead to joint space reduction, followed by disc compression, and resulting pain in the masticatory muscles. Occlusion is treated not only as the ratio of contact between teeth, but also as a dynamic, morphological and functional relation, presenting a great influence on chewing, swallowing and speech [9]. Tooth wear alters the existing occlusal plane introducing deflective occlusal interferences [2]. Occlusal/skeletal factors, such as crossbite, overjet and centric relation (CR) to maximum intercuspation (MI) discrepancy greater than 2 mm could be considered occlusal risk factors for TMD [6]. These are of serious concern to the patient and the dental professionals because of its relation to the temporo mandibular joint disorders. Therefore, it is of interest to evaluate the association of occlusal wear facets in TMD patients.

## Materials and methods:

### Study designs and Study setting:

The present study was conducted in a university setting (Saveetha dental college and hospitals, Chennai, India). Thus the data available is of patients from the same geographic location and have similar ethnicity. The retrospective study was carried out with the help of digital case records of 98 patients who reported to the hospital. Ethical clearance to conduct this study was obtained from the Scientific Review Board of the hospital. The ethical approval number for the present study is SDC/SIHEC/2020/DIASDATA/0619-0320.

### Sampling:

98 patients were reviewed from patient's records and data were extracted. Only relevant data was included to minimize sampling bias. Simple random sampling method was carried out. Cross verification of data for error was done by presence of additional reviewer and by photographic evaluation.

### Data Collection:

A single calibrated examiner evaluated the digital case records of patients who reported to Saveetha Dental College from June 2019 to March 2020. For the present study, inclusion criteria were data of patients with TMD and patients without TMD having no systemic diseases and records of these patients with incomplete data were excluded from the study. Clinical examination, dental status and photographs of these patients' records were reviewed. Grading was done using, Smith and Knight tooth wear index (TWI) [10] for evaluation of occlusal wear facets based on extent to which teeth wear was present. The gradings are as follows:

Grade 0: No teeth wear

Grade 1: Loss of enamel surface characteristics

Grade 2: Teeth wear of less than 1/3rd of the tooth

Grade 3: Teeth wear causing dentin exposure for more than 1/3rd teeth wear

Grade 4: Pulpal exposure due to teeth wears

### Statistical analysis:

The collected data was tabulated and analysed with Statistical Package for Social Sciences for Windows, version20.0 (SPSS Inc., Chicago, IL, USA) and results were obtained. Categorical variables were expressed in frequency and percentage. Chi square test was used to test association between categorical variables. Chi square tests were carried out using age, gender and occlusal wear facets as independent variables and temporo mandibular disorder as dependent variable. The statistical analysis was done by pearson chi square test. P value < 0.05 was considered statistically significant.

## Results:

Out of 49 patients with TMD, 46.9% of them belonged to the age group of 26-40 years, 30.6% belonged to the age group below 25 years, 16.3% belonged to the age group of 41-55 years and 6.2% belonged to the age group above 55 years (Figure 1 - see PDF). Majority of the patients with TMD were in the age group of 26-40 years (Table 1 - see PDF). 26 (53.1%) out of 49 TMD patients were females and 23 (46.9%) were males (Figure 2 - see PDF). It is thus evident that TMD was present more in females compared with males (Table 2 - see PDF). On comparison of Figure 3 and Figure 4 (see PDF), it was observed that in the age group below 25 years in TMD patients, 33.4% had grade1 and 6.6% had grade2 (p value-0.034 (p<0.05), hence statistically significant) while in the same age group, patients without TMD had no teeth wear and grade0 was only observed (pvalue-0.00 (p<0.05), hence statistically significant). In the age group of 26-40years, patients with TMD showed 30.4% grade 1, 13.1% grade2 and 4.4% grade4 while in the same age group of patients without TMD majority did not show teeth wear and 21.1% had grade1. Grade 2 (62.5%) was the most prevalent teeth wear grade in 41-55 years of age in TMD patients while the same age group patients without TMD also showed higher prevalence of grade 2(50%). However, in the age group of above 55 years, the groups showed high prevalence of teeth wear of which, 66.7% of the patients with TMD had grade2 and in patients without TMD, 75% had grade 2. On comparison of Figure 5 and Figure 6 (see PDF), it was observed that in patients with TMD, 43.5% of males and 65.4% of females had teeth wear (p value- 0.34 (p>0.05), hence statistically not significant), while in patients without TMD, 40.8% of the males and only 36.4% of the females showed presence of teeth wear (p value- 0.823 (p>0.05), hence statistically not significant). It was observed from Figure 7(see PDF) that higher teeth wear grades were prevalent in TMD patients compared to patients without TMD (p value- 0.340 (p>0.05), hence statistically not significant). Teeth wear grade1 was present in 51.8% of TMD patients and 48.2% in patients without TMD; grade2 was present in 68.7% of TMD patients and 31.3% in patients without TMD. Grade3 was present equally in both the groups and grade 4 was observed only in TMD patients. Teeth wear grade2 showed the highest prevalence among patients with TMD. Posterior teeth wear was observed more in TMD patients (61.%) while generalised teeth wear was more in patients without TMD (57.2%)(p value-0.178 (p>0.05), hence statistically not significant) (Figure 8 - see PDF). Overall it was observed that 55.1% of the patients with TMD had teeth wear and 38.8% of patients without TMD had teeth wear. Chi square test was done and association between age group and teeth wear grade was found to be statistically significant (P<0.05). While association between gender, TMD and teeth wear was statistically not significant (P>0.05).

## Discussion:

Thus, from this study it was observed that teeth wear was present more in patients with TMD (55%). Study population showed teeth wear was more prevalent in the age group of 26-40 years. Also, females showed more prevalence of teeth wear than males. Most patients with TMD showed posterior teeth wear than generalised teeth wear. Similar to the present study, Costa et al. [11] reported a high incidence of teeth with wear facets, observed in patients with TMD dysfunction. Few authors as well observed similar results in their study [4,12]. Increased prevalence of teeth wears in TMD patients were attributed to high incidence of grinding and clenching habits in these patients and due to the obvious connection between the structures of temporo mandibular joint and the dental occlusion [13]. In accordance to the present study, Luther et al. [14] also reported that increased teeth wear in TMD patients was reported in younger age group, while few authors stated no association between age and teeth wear was present [15,16]. As in this study 26-40 years showed higher incidence of teeth wear in TMD patients, Renecker et al. [17] as well reported similar findings.

They suggested that severe teeth wear is mostly the result of bruxing activity and to a lesser extent as a result of functional wear. Increased teeth wear was observed in females than males in the present as well as in studies done by other authors [11,14,18]. According to Luther [14] the high incidence in women may be related to hormonal changes that occur at this stage of life.In addition, psychological factors and increased levels of somatization, that is, depression or anxiety have been closely linked to TMD which may also be the reason why females are more affected [18]. In contrast, Yadav [19] and Solberg et al. [20] reported that males showed increased teeth wear compared to females and attributed this to strong masseter function and greater muscle fiber mass. As observed in the present study that posterior teeth wear was more in TMD patients, several other studies [15] also reported that the posterior teeth wear was more in TMD patients. They also stated that these changes are associated with joint changes, particularly increasing the risk of cracking, and disc displacement. The occlusal instability caused by the changes in posterior teeth may cause TMD since the occlusal changes; muscle changes and joint changes exceed the adaptive threshold of the stomatognathic system. The early rehabilitation treatment in asymptomatic patients may be indicated in order to prevent the occlusal collapse and, consequently, to reduce the risk of future joint problems. In the past, studies have suggested that occlusal interferences were main factors in TMD development, thus, validating irreversible occlusal therapies as the definitive treatment of the disorder [18]. Based on that, occlusal adjustments, full mouth rehabilitation and orthodontic treatment became very popular as the treatment of choice for TMD. This study could pave way for future research to be done for better understanding of the signs and symptoms of TMD and aid in early intervention and better treatment modalities for improved results in clinical practice. The limitations of the study include limited sample size and limited demographics.

## Conclusion

Data shows that teeth wear was present more in patients with TMD (55%). The age group 26-40 years showed high prevalence of teeth wear (grade 1) in TMD patients (P value = 0.034). TMD was present more in females than males. Female (54%) patients with TMD showed more teeth wear compared to males. Most patients with TMD showed posterior teeth wear (61%) than generalized teeth wear. Thus, association was present between occlusal teeth wear and TMD patients especially in the age group of 26-40 years. Hence, proper evaluation of occlusal factors will aid in early diagnosis of TMDs.

## Clinical significance:

The study helps us to understand the manifestation of occlusal teeth wear in patients with temporo mandibular disorder.

## References

[1] Huang GJ (2002). J Dent Res.

[2] https://www.elsevier.com/books/management-of-temporomandibular-disorders-and-occlusion/okeson/978-0-323-08220-4.

[3] Magnusson T (2005). Acta Odontologica Scandinavica.

[4] Fujita Y (2003). Bull Tokyo Dent Coll.

[5] Ishigaki S (1992). Cranio.

[6] https://www.academia.edu/23447841/Functional_Occlusion_From_TMJ_to_Smile_Design_2007_.

[7] Poveda-Roda R (2013). Medicina Oral Patología Oral y Cirugia Bucal.

[8] Cairns BE (2010). Journal of Oral Rehabilitation.

[9] Gesch D (2004). Angle Orthod.

[10] Lopez-Frias FJ (2012). Journal of Clinical and Experimental Dentistry.

[11] Costa MD (2012). Dental Press Journal of Orthodontics.

[12] Pergamalian A (2003). J Prosthet Dent..

[13] John MT (2005). Pain.

[14] Luther F (2007). British Dental Journal.

[15] Seligman DA (1988). Journal of Dental Research.

[16] Clarke NG (1984). Journal of Oral Rehabilitation.

[17] Reneker J (2011). J Orthop Sports Phys Ther.

[18] Henrikson T, Nilner M (2003). Journal of Orthodontics.

[19] Yadav S (2011). The Journal of Indian Prosthodontic Society.

[20] Solberg WK (1972). The Journal of Prosthetic Dentistry.

